# Can Artificial Intelligence and Machine Learning Transform Prediction and Treatment of Post-Transjugular Intrahepatic Portosystemic Shunt (TIPS) Overt Hepatic Encephalopathy?

**DOI:** 10.1016/j.gastha.2024.09.015

**Published:** 2024-09-30

**Authors:** Eric Kalo, Scott Read, Jacob George, Avik Majumdar, Golo Ahlenstiel

**Affiliations:** Blacktown Clinical School, School of Medicine, Western Sydney University, Penrith, New South Wales, Australia; Blacktown Mount Druitt Hospital, WSLHD, Blacktown, New South Wales, Australia; Blacktown Clinical School, School of Medicine, Western Sydney University, Penrith, New South Wales, Australia; Blacktown Mount Druitt Hospital, WSLHD, Blacktown, New South Wales, Australia; Storr Liver Centre, The Westmead Institute for Medical Research, Westmead, New South Wales, Australia; Storr Liver Centre, The Westmead Institute for Medical Research, Westmead, New South Wales, Australia; Westmead Hospital, WSLHD, Westmead, New South Wales, Australia; Victorian Liver transplant Unit, Austin Health, Heidelberg, Victoria, Australia; The University of Melbourne, Parkville, Victoria, Australia; Blacktown Clinical School, School of Medicine, Western Sydney University, Penrith, New South Wales, Australia; Blacktown Mount Druitt Hospital, WSLHD, Blacktown, New South Wales, Australia; Storr Liver Centre, The Westmead Institute for Medical Research, Westmead, New South Wales, Australia

Over the past decade, artificial computational systems in particular artificial intelligence (AI) and derived technologies have become increasingly integrated into clinical practice, revolutionizing care, outperforming/surpassing humans for certain medical tasks, and driving precision and personalized medicine. This transformation has accelerated with the launch of platforms such as “Chat-GPT” that opened new avenues for the implementation of AI systems into clinical practice.

AI comprises computer algorithms that can mimic human cognitive functions.^1^ It encompasses overlapping disciplines including machine learning (ML) and deep learning (DL) (Figure). ML is the method to learn from data or experience that requires data curation including classification and feature selection. DL uses larger datasets and significant computing power but does not require significant data curation prior to analysis. DL utilizes a multilayer artificial neural network (ANNs) similar to the architecture of biological neural networks to interpret and classify data while adapting as the data passes through multiple layers of the algorithm.^2^ While both ML and DL can achieve high prognostic performance, DL models are inherently more accurate than traditional ML models.

**Figure:**
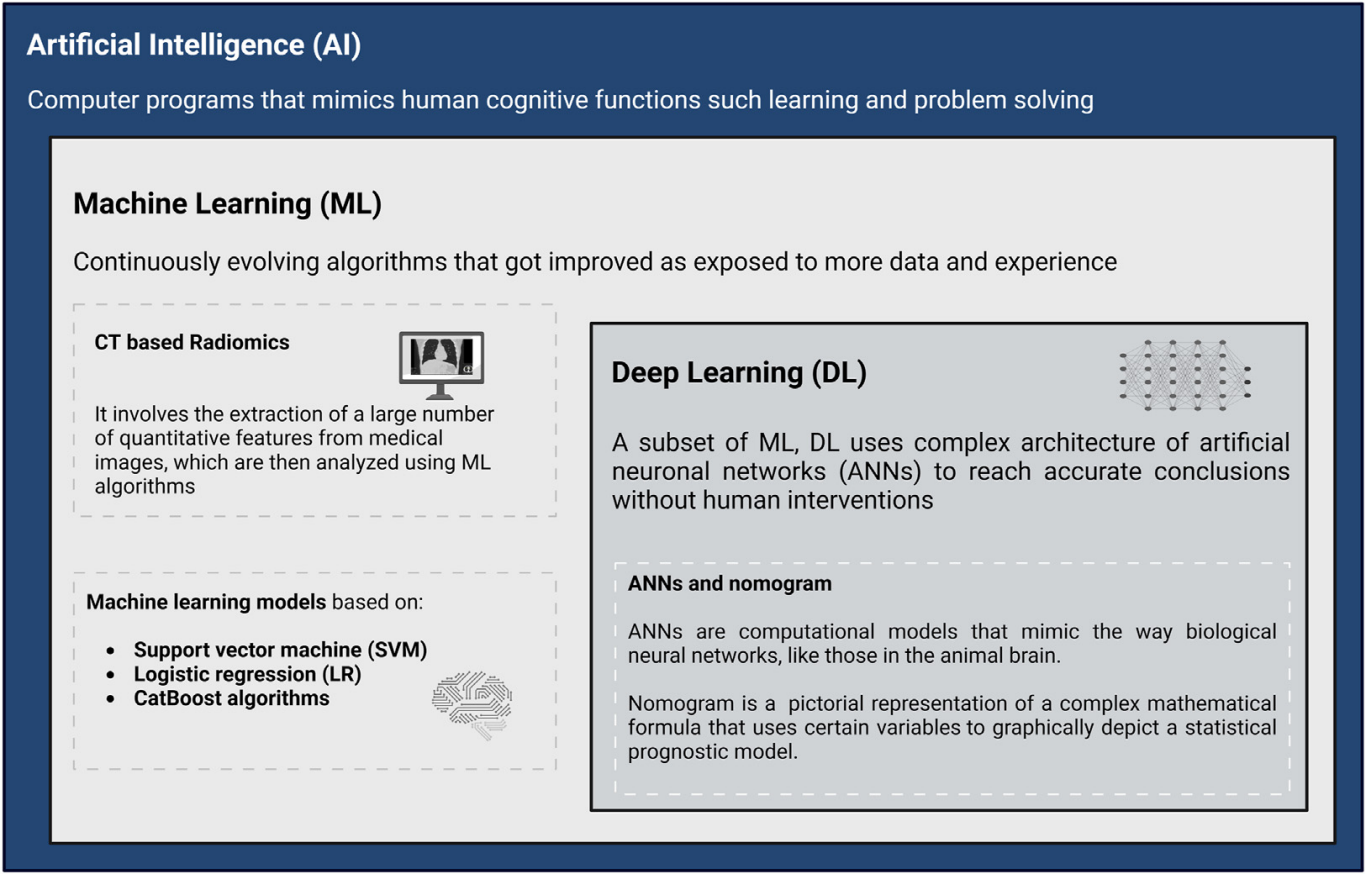
Schematic illustration of the hierarchy of artificial intelligence and its machine learning and deep learning disciplines. Dotted squares represent proposed AI models for prediction of post-TIPS HE.

Transjugular intrahepatic portosystemic shunt (TIPS) is one of the most technically challenging interventional radiological procedures used to treat the complications of portal hypertension. Despite careful patient selection for TIPS and search for encephalopathy prior to TIPS placement, the onset of a new episode or worsening of pre-existing hepatic encephalopathy (HE) post-TIPS is common. An episode of overt HE can occur in up to 30%–50% of patients post-TIPS.^3^, ^4^, ^5^

Numerous factors have been identified to confer an increased risk for the development of post-TIPS HE, including older age, severe liver disease (Child- Turcotte-Pugh class C, Child-Pugh Score ≥10, or model for end-stage liver disease score >18), history of prior HE, shunt diameter, sarcopenia, elevated creatinine, hyponatremia, and low portosystemic pressure gradient (PSPG) post-TIPS placement.^6^, ^7^, ^8^

All current methods of psychometric evaluation of HE, be it the animal naming test or computer-assisted approaches, carry major shortcomings and inherent biases owing to the learning effect from repeated testing, the influence of baseline patient intelligence and age.^9^ Currently, there are no decision tools in practice that are universally accepted for the prediction of post-TIPS HE. ML-based AI models have been proposed in the wake of the “AI revolution” to predict HE following TIPS. The hope is to develop computational tools that supplement current risk assessment techniques and inform prognosis and management. Here, we report on AI-based tools that are poised to reshape post-TIPS HE prediction and treatment from radiomics to DL-ANNs, nomograms, and ML algorithms (Table). Furthermore, we suggest conceptual algorithms that can refine post-TIPS HE prediction.

**Table:** Prediction Modalities for Post-TIPS Hepatic Encephalopathy

Prediction modality for post-TIPS HE	Pros	Cons
CT radiomic of right lobe of liver or visceral adipose tissue	• Highly accurate • Noninvasive • Can be automated to reduce time and interobserver variability • Extraction of quantitative features that are not accessible through human perception alone	• Requires high-quality images • Manual annotation, not available in usual clinical setting • Limited sample size and lack of external validations • Cannot predict the severity and time to occurrence of HE
Machine learning models based on support vector machine (SVM), logistic regression (LR), and CatBoost algorithms	• Models built using clinical, laboratory, and procedural data • Models can significantly outperform linear regression models	• Retrospective design, selection bias • Can determine early onset of post-TIPS HE (≤1 month) • Model included institution specific procedural features
Artificial neural network and nomogram model	• Accurately predicts early occurrence of HE, mortality, and liver dysfunction after early TIPS creation for patients with cirrhosis and AVB • Incomplete data sets can be used for predictions • Deep learning models are inherently more accurate than machine learning	• Retrospective design • Lack of external validation of the model • High computational requirements • The decision-making process of ANNs can be opaque, resulting in difficulty interpreting conclusions (black box) • Overfitting and lack of explainability

AVB, acute variceal bleeding.

A potential AI-integrated tool for post-TIPS HE prediction is radiomics. Radiomics is a process that extracts high-throughput quantitative and high-dimensional mineable data from radiological images (eg, spatial distribution and signal intensities) and applies advanced data characterization algorithms to produce a prediction. Radiomics has a remarkable ability to capture mesoscopic features that are not readily observable to the human eye. Cheng *et al.* extracted radiomic features from routinely acquired pre-TIPS CT images of the right lobe of the liver, and using random forest models, could outperform traditional clinical parameter-based models (linear regression models) in the prediction of post-TIPS overt HE (*P* value < .05).^10^ The radiomics model displayed favorable performance in the test cohort with an area under the curve (AUC) of 0.887 (95% confidence interval, 0.760–1.00) with an accuracy of 90.9%, a sensitivity of 78.6%, and a specificity of 100%. Conversely, the test cohort of the clinical model had an AUC of 0.606 (95% confidence interval, 0.511–0.701). The accuracy of the clinical model in the test group was 66.7% with a sensitivity of 78.6% and a specificity of 58.9%. Remarkably, the combination of independent clinical predictors and radiomic features in a single model did not increase the predictive value.

Recently, the same group evaluated the performance and clinical utility of CT radiomic features of visceral adipose tissue in the prediction of post-TIPS HE.^11^ Although the relationship between nutritional status and post-TIPS HE may be more complex,^12^ the group has demonstrated that ten radiomic features and C-reactive protein constituting the radiomic-clinical models had satisfactory performance with an average AUC of 0.84. The model can putatively provide 90% sensitivity and 100% negative predictive value. Of note, the model did not take into consideration the gender differences in adipose tissue distribution that may affect its performance. In addition, the radiomic model cannot predict the severity and time to occurrence of HE post-TIPS.

Similarly, magnetic resonance-based radiomic features for diagnosing chronic HE and grading its severity were explored by an Italian group.^13^ This group combined textural features extracted on T1-weighted imaging on bilateral lentiform nuclei at the level of the Foramen of Monro. The radiomic-based model was highly accurate in predicting cirrhosis AUC of 0.97 and moderately accurate in predicting the presence of grade ≥2 chronic HE (AUC of 0.82). However, the group did not evaluate the performance of magnetic resonance-based radiomics in patients considered for TIPS.

Another use of DL for post-TIPS HE prediction is the ANNs and nomogram models explored by Zhong et al. in 207 patients at 3-month follow-up after TIPS .^14^ The models developed based on independent risk factors such as ALBI (Albumin-Bilirubin) grade, sex, age, and presence of alcohol-related liver cirrhosis, all of which were shown to be significant in a univariate analysis for the prediction of HE. These results show that such models have the potential to accurately predict early occurrence of overt HE (within 3 months) and liver dysfunction post-TIPS for patients with cirrhosis and acute variceal bleeding. The ANN models predicting overt HE demonstrated an AUC of 0.816. Unfortunately, this study did not analyze the association between hepatic vein pressure gradient and post-TIPS HE.

A recent retrospective study included three different ML-based AI models developed using clinical, laboratory, and procedural features in 327 patients who underwent TIPS placement.^15^ The models were built using support vector machine, logistic regression, and CatBoost algorithms and were validated by the five-fold nested cross-validation method. In the univariable analysis of the study, a higher post-TIPS PSPG was associated with a higher likelihood of HE (*P* = .004). However, the PSPG was not selected by a wrapper-based sequential feature selection algorithm. The wrapper-based sequential feature selection algorithm assessed the performance of all features through its own estimator and selected seven features. Among these features, variceal bleeding, creatinine, indirect bilirubin, and sodium model for end-stage liver disease have been proposed as predictors for occurrence of postprocedural HE. The support vector machine, logistic regression, and CatBoost algorithms demonstrated good accuracy in predicting the onset of post-TIPS HE (74%, 75%, and 73%, respectively). Although the three algorithm-based models work on different principles, the AUC values were 0.82, 0.83, and 0.83, respectively. One of the major limitations of this study is the incorporation of institution-specific procedural aspects. These aspects may in turn challenge the utility of the models for other patient cohorts.

Despite the promising results, future research is still needed to identify the ideal predictors of post-TIPS HE. The advantage of ML is its vast data processing potential and its ability to learn from complex and diverse clinical datasets. AI algorithms for post-TIPS HE could be further refined by integrating parameters such as a left portal venous puncture. A randomized trial of 72 patients demonstrated that patients undergoing TIPS through the left branch of the portal vein had a lower incidence of de novo encephalopathy when compared to those who had TIPS via a right branch of the portal vein (*P* = .036 and .012 respectively).^16^

Another conceptual algorithm that can be proposed for post-TIPS HE prediction is mapping features characterizing the underlying patterns of speech or handwriting of patients with HE using signal processing algorithms. Studies have shown that patients with overt HE typically have an impaired speech rate and precision.^17^^,^^18^ Data acquired from speech and handwriting can be obtained at low cost with minimal patient distress. For this approach, a trained convoluted neural network, a class of multilayered DL neural networks, would require the use of a simple smartphone only. Other innovative methods that can be adopted to refine prediction include novel spectral electroencephalography thresholds to optimize electroencephalography performance and examination of regional homogeneity of brain intrinsic activity for HE diagnosis.^19^^,^^20^ Recently, Dieta-App offered better insights into the dose of lactulose and HE treatment by evaluating AI-based Bristol stool scale with stool images.^21^ Such examples of AI tools may enhance HE management and prove useful for patients post-TIPS procedure.

AI has offered promising avenues for the prediction and management of post-TIPS HE. Despite the exciting potential for AI-based models to improve clinical care following TIPS placement, there remain significant challenges to translate these potentially transformative technologies from research to practice. Currently available datasets are limited by both their modest cohort sizes and derivation from single-center studies. Large, multicenter studies are required to evaluate AI-based prediction models in the context of real-world variability resulting from differences in models of practice and patient cohorts. Other challenges that need to be addressed span from the economic to technical aspects such as ensuring the absence of flawed algorithms, proof of concept validation in randomized clinical trials across countries, to legal and ethical issues, racial bias, and data security.
